# The genome sequence of Daubenton’s bat, *Myotis daubentonii *(Kuhl, 1817)

**DOI:** 10.12688/wellcomeopenres.21081.1

**Published:** 2024-03-01

**Authors:** Manuel Ruedi, Sonja C. Vernes, Emma C Teeling, Meike Mai, Darwin Tree of Life Barcoding collective, Wellcome Sanger Institute Tree of Life Management, Samples and Laboratory team, Wellcome Sanger Institute Scientific Operations: Sequencing Operations, Wellcome Sanger Institute Tree of Life Core Informatics team, Tree of Life Core Informatics collective, Darwin Tree of Life Consortium

**Affiliations:** 1 Muséum d'histoire naturelle de Genève, Geneva, Switzerland; 2 School of Biology, University of St Andrews, St Andrews, Scotland, UK; 3 School of Biology and Environmental Science, University College Dublin, Dublin, Leinster, Ireland

**Keywords:** Myotis daubentonii, Daubenton's bat, genome sequence, chromosomal, Chiroptera

## Abstract

We present a genome assembly from an individual male *Myotis daubentonii* (Daubenton's bat; Chordata; Mammalia; Chiroptera; Vespertilionidae). The genome sequence is 2,127.8 megabases in span. Most of the assembly is scaffolded into 23 chromosomal pseudomolecules, including the X and Y sex chromosomes. The mitochondrial genome has also been assembled and is 17.34 kilobases in length.

## Species taxonomy

Eukaryota; Metazoa; Eumetazoa; Bilateria; Deuterostomia; Chordata; Craniata; Vertebrata; Gnathostomata; Teleostomi; Euteleostomi; Sarcopterygii; Dipnotetrapodomorpha; Tetrapoda; Amniota; Mammalia; Theria; Eutheria; Boreoeutheria; Laurasiatheria; Chiroptera; Yangochiroptera; Vespertilionoidea; Vespertilionidae; *Myotis* Kaup, 1829 (subordinal taxonomy updated per Teeling *et al.* (2005); *Myotis daubentonii* (Kuhl, 1817) (NCBI:txid98922)

## Background

**Figure 1.**
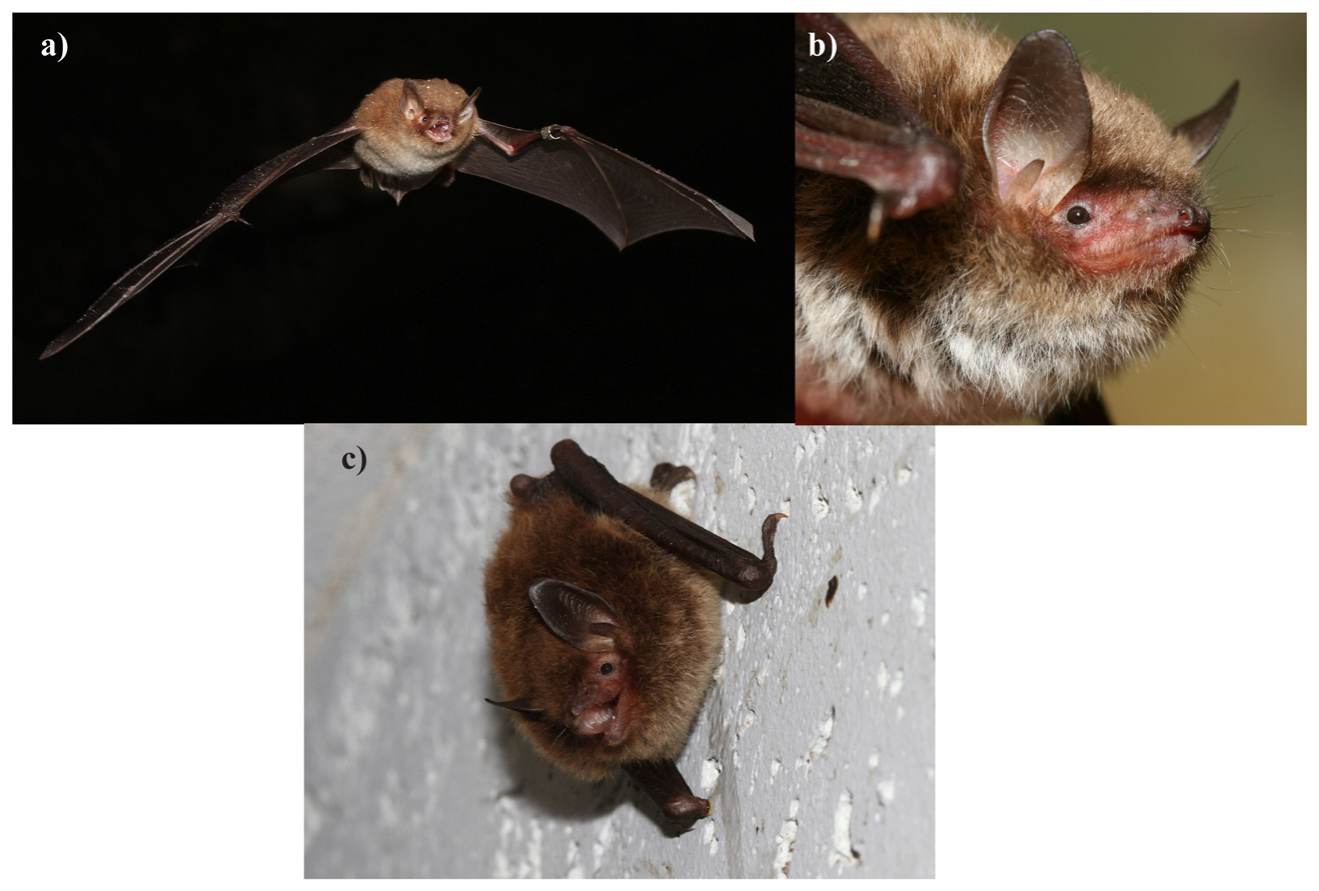
Photographs of *Myotis daubentonii* by Manuel Redi (not the specimen used for genome sequencing).

Daubenton’s bats *Myotis daubentonii* (Kuhl, 1817) are small bats weighing 6–10 g and with a forearm length of 35–42 mm. Their dense, brownish dorsal fur contrasts with a whitish belly but their face (especially around eyes) is distinctively naked ( Figure 1). They have relatively short ears and tragus. Their feet are long, making up more than 60% of their tibia length, which, together with their broad uropatagium, enables them to hunt aquatic insects over calm waters of rivers, canals, ponds, or lake shores. Daubenton’s bats usually roost in hollow trees, where females form nursery colonies of typically 20 to 80 individuals. They are known to often change roost during the nursing season. Males also roost in trees or under bridges but occupy different, less productive habitats than those preferred by the females ( Linton & Macdonald, 2019). During the autumnal mating season, they engage in a swarming behaviour, where males from several species aggregate near the entrance of underground roosts to attract females ( Glover & Altringham, 2008; Parsons *et al.*, 2003). These bats hibernate in natural or man-made undergrounds such as caves, tunnels, or mines.

The Daubenton’s bat, a common and widespread European species is classified as Least Concern by the IUCN ( Kruskop *et al.*, 2021). Its new complete genome will be of high interest as *M. daubentonii* is associated with European lyssavirus 2 ( Atterby *et al.*, 2010), including in the Lake Geneva region ( Megali *et al.*, 2010), where the specimen used for genome sequencing originates.

Phylogenetic reconstructions place *M. daubentonii* within the Old World radiation of *Myotis* as a sister-species to the distinctive Bechstein’s bat ( Ruedi *et al.*, 2013). However, reconstructions based on different regions of the genome (i.e. mitochondrial versus nuclear markers) provide conflicting phylogenetic perspectives ( Foley *et al.*, 2024; Morales *et al.*, 2019). This new, complete genome associated with comparable *Myotis* genomes (e.g. Jebb *et al.*, 2020), will thus provide new clues to better understand the evolution of this remarkable world-wide radiation.

## Genome sequence report

The genome was sequenced from one male *Myotis daubentonii* collected from Cologny, Geneva, Switzerland (see Methods). A total of 40-fold coverage in Pacific Biosciences single-molecule HiFi long reads was generated. Primary assembly contigs were scaffolded with chromosome conformation Hi-C data. Manual assembly curation corrected 48 missing joins or mis-joins and removed one haplotypic duplications, reducing the assembly length by 0.43% and the scaffold number by 25.15%, and increasing the scaffold N50 by 5.74%.

**Figure 2.**
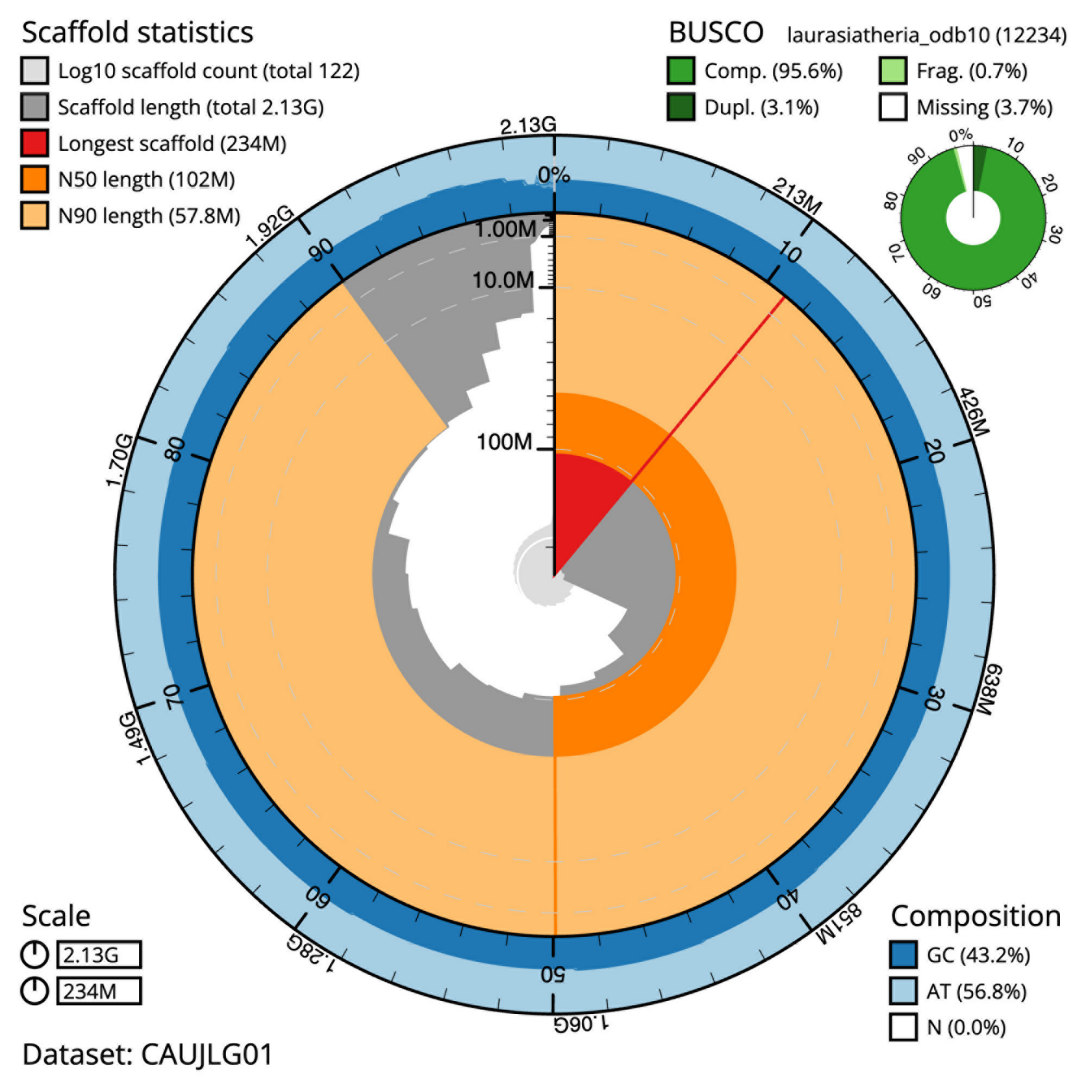
Genome assembly of *Myotis daubentonii*, mMyoDau2.1: metrics. The BlobToolKit snail plot shows N50 metrics and BUSCO gene completeness. The main plot is divided into 1,000 size-ordered bins around the circumference with each bin representing 0.1% of the 2,127,824,474 bp assembly. The distribution of scaffold lengths is shown in dark grey with the plot radius scaled to the longest scaffold present in the assembly (234,112,155 bp, shown in red). Orange and pale-orange arcs show the N50 and N90 scaffold lengths (102,234,773 and 57,790,901 bp), respectively. The pale grey spiral shows the cumulative scaffold count on a log scale with white scale lines showing successive orders of magnitude. The blue and pale-blue area around the outside of the plot shows the distribution of GC, AT and N percentages in the same bins as the inner plot. A summary of complete, fragmented, duplicated and missing BUSCO genes in the laurasiatheria_odb10 set is shown in the top right. An interactive version of this figure is available at https://blobtoolkit.genomehubs.org/view/CAUJLG01/dataset/CAUJLG01/snail.

**Figure 3.**
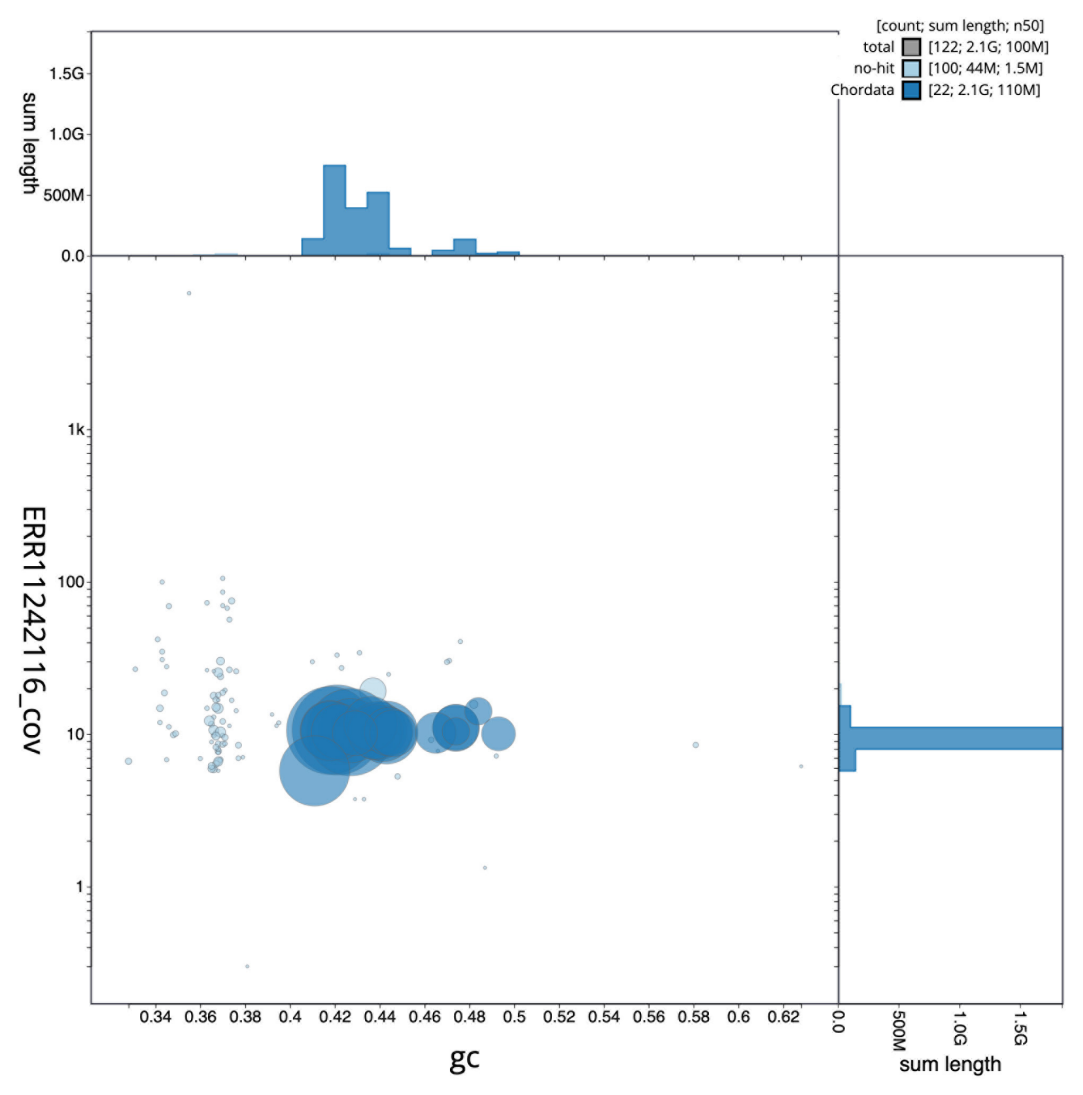
Genome assembly of *Myotis daubentonii*, mMyoDau2.1: BlobToolKit GC-coverage plot. Sequences are coloured by phylum. Circles are sized in proportion to sequence length. Histograms show the distribution of sequence length sum along each axis. An interactive version of this figure is available at https://blobtoolkit.genomehubs.org/view/CAUJLG01/dataset/CAUJLG01/blob.

**Figure 4.**
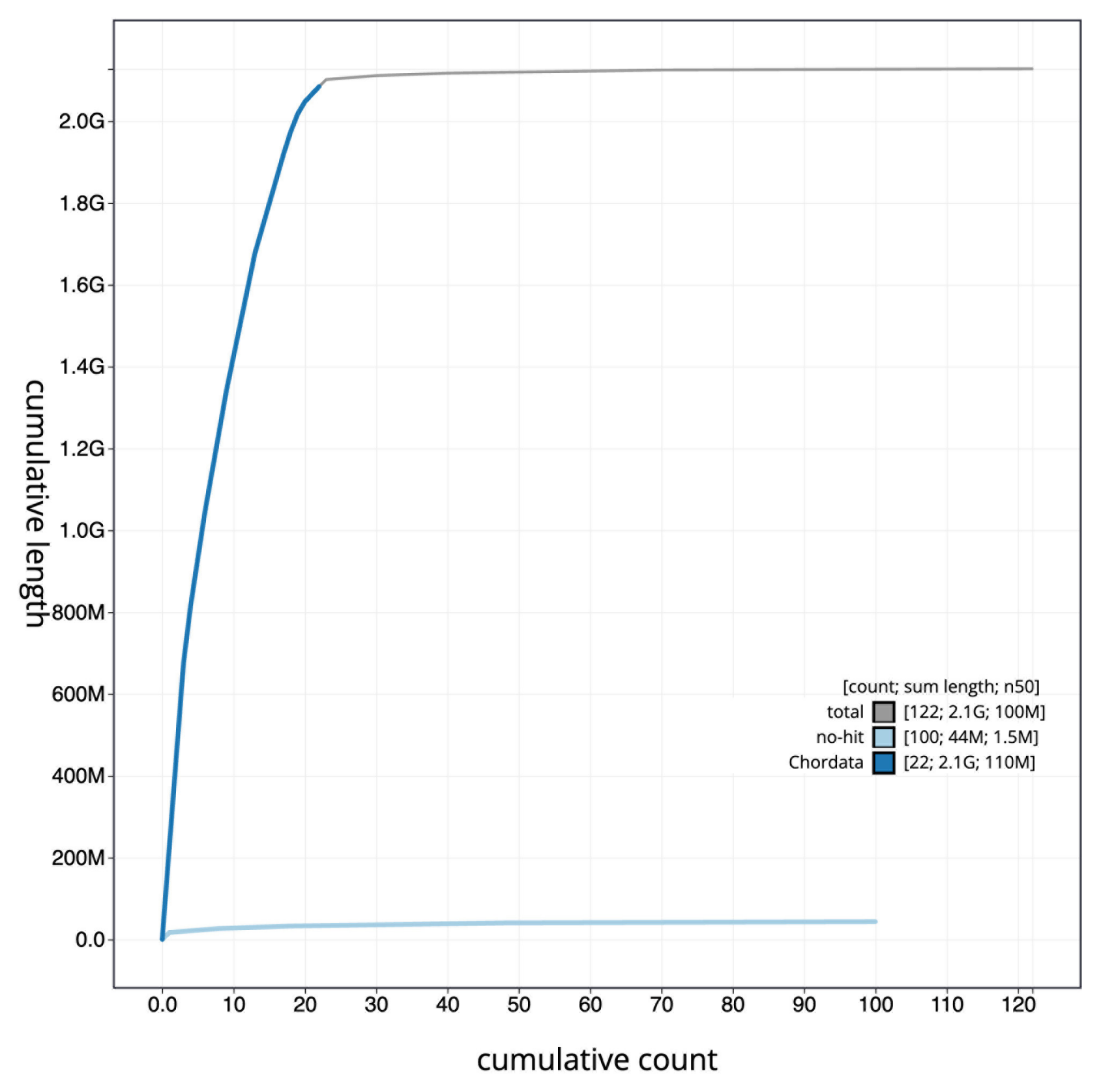
Genome assembly of *Myotis daubentonii*, mMyoDau2.1: BlobToolKit cumulative sequence plot. The grey line shows cumulative length for all sequences. Coloured lines show cumulative lengths of sequences assigned to each phylum using the buscogenes taxrule. An interactive version of this figure is available at https://blobtoolkit.genomehubs.org/view/CAUJLG01/dataset/CAUJLG01/cumulative.

**Figure 5.**
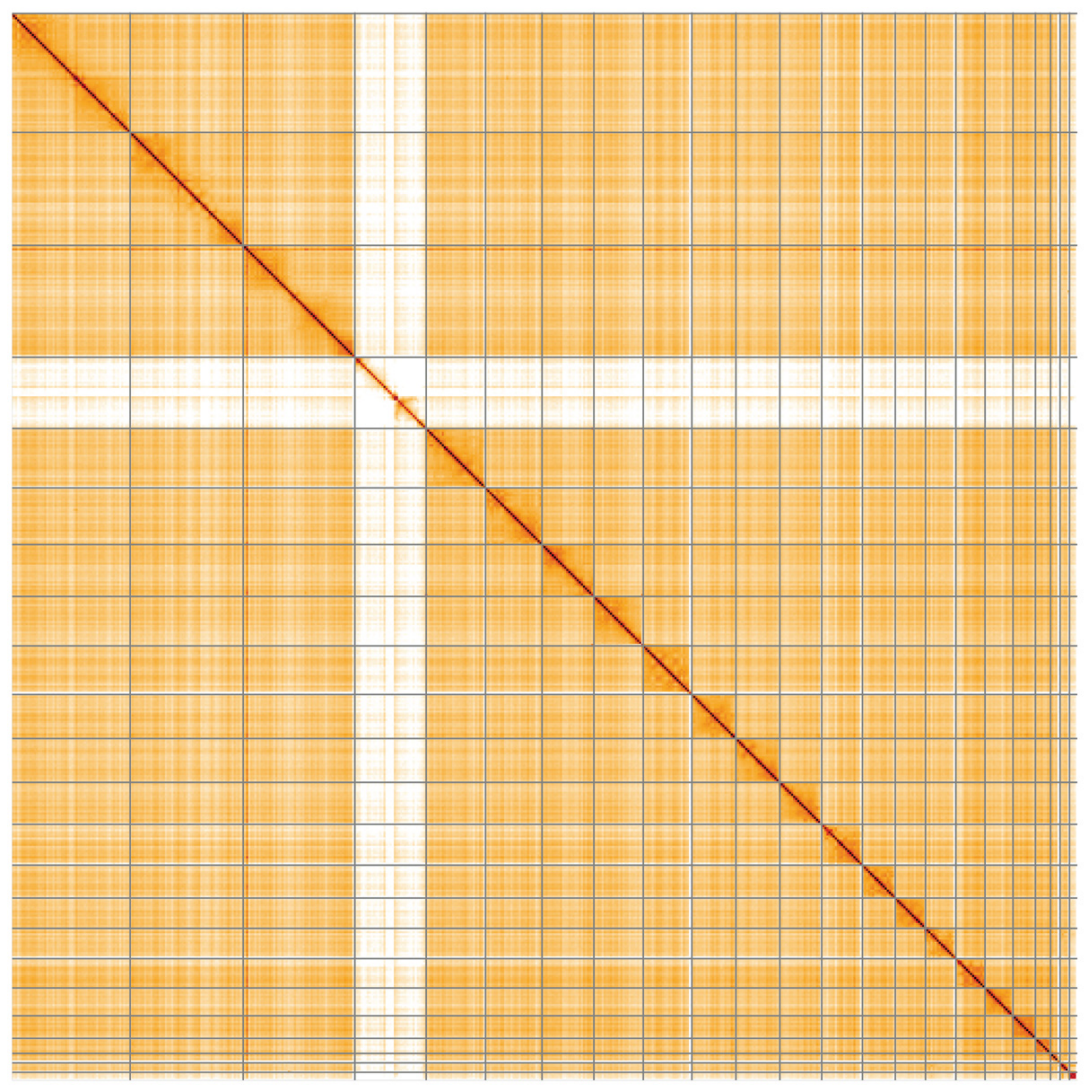
Genome assembly of *Myotis daubentonii*, mMyoDau2.1: Hi-C contact map of the mMyoDau2.1 assembly, visualised using HiGlass. Chromosomes are shown in order of size from left to right and top to bottom. An interactive version of this figure may be viewed at https://genome-note-higlass.tol.sanger.ac.uk/l/?d=R61mZBFdSo22y-Q-aB7tVw.

The final assembly has a total length of 2,127.8 Mb in 121 sequence scaffolds with a scaffold N50 of 102.2 Mb ( Table 1). The snail plot in Figure 2 provides a summary of the assembly statistics, while the distribution of assembly scaffolds on GC proportion and coverage is shown in Figure 3. The cumulative assembly plot in Figure 4 shows curves for subsets of scaffolds assigned to different phyla. Most (98.8%) of the assembly sequence was assigned to 23 chromosomal-level scaffolds, representing 21 autosomes and the X and Y sex chromosomes. Chromosome-scale scaffolds confirmed by the Hi-C data are named in order of size ( Figure 5; Table 2). While not fully phased, the assembly deposited is of one haplotype. Contigs corresponding to the second haplotype have also been deposited. The mitochondrial genome was also assembled and can be found as a contig within the multifasta file of the genome submission.

**Table 1.** Genome data for *Myotis daubentonii*, mMyoDau2.1.

Project accession data		
Assembly identifier	mMyoDau2.1	
Species	*Myotis daubentonii*	
Specimen	mMyoDau2	
NCBI taxonomy ID	98922	
BioProject	PRJEB61137	
BioSample ID	SAMEA9921455	
Isolate information	mMyoDau2, male: heart and muscle tissue (DNA, Hi-C and RNA sequencing)	
Assembly metrics *		*Benchmark*
Consensus quality (QV)	60.3	*≥ 50*
*k*-mer completeness	100.0%	*≥ 95%*
BUSCO **	C:95.6%[S:92.5%,D:3.1%], F:0.7%,M:3.7%,n:12,234	*C ≥ 95%*
Percentage of assembly mapped to chromosomes	98.8%	*≥ 95%*
Sex chromosomes	XY	*localised homologous pairs*
Organelles	Mitochondrial genome: 17.34 kb	*complete single alleles*
Raw data accessions		
PacificBiosciences SEQUEL II	ERR11242116, ERR11809131, ERR11242114, ERR11242115	
Hi-C Illumina	ERR11217108	
PolyA RNA-Seq Illumina	ERR11217109	
Genome assembly		
Assembly accession	GCA_963259705.1	
*Accession of alternate haplotype*	GCA_963242275.1	
Span (Mb)	2,127.8	
Number of contigs	1,221	
Contig N50 length (Mb)	3.1	
Number of scaffolds	121	
Scaffold N50 length (Mb)	102.2	
Longest scaffold (Mb)	234.98	

* Assembly metric benchmarks are adapted from column VGP-2020 of “Table 1: Proposed standards and metrics for defining genome assembly quality” from Rhie *et al.* (2021). ** BUSCO scores based on the laurasiatheria_odb10 BUSCO set using version 5.3.2. C = complete [S = single copy, D = duplicated], F = fragmented, M = missing, n = number of orthologues in comparison. A full set of BUSCO scores is available at https://blobtoolkit.genomehubs.org/view/CAUJLG01/dataset/CAUJLG01/busco.

**Table 2.** Chromosomal pseudomolecules in the genome assembly of *Myotis daubentonii*, mMyoDau2.

INSDC accession	Chromosome	Length (Mb)	GC%
OY725359.1	1	234.11	42.0
OY725360.1	2	222.57	42.0
OY725361.1	3	220.08	42.5
OY725363.1	4	116.92	42.5
OY725364.1	5	111.43	44.5
OY725365.1	6	102.23	42.0
OY725366.1	7	97.17	42.0
OY725367.1	8	95.74	44.0
OY725368.1	9	86.66	44.0
OY725369.1	10	86.43	42.0
OY725370.1	11	82.56	44.0
OY725371.1	12	80.36	43.5
OY725372.1	13	64.61	44.5
OY725373.1	14	59.7	44.5
OY725374.1	15	59.55	47.5
OY725375.1	16	57.79	47.5
OY725376.1	17	54.85	43.0
OY725377.1	18	44.3	46.5
OY725378.1	19	29.7	49.5
OY725379.1	20	18.7	47.5
OY725380.1	21	18.48	48.5
OY725362.1	X	140.35	41.0
OY725381.1	Y	17.19	43.5
OY725382.1	MT	0.02	35.5

The estimated Quality Value (QV) of the final assembly is 60.3 with *k*-mer completeness of 100.0%, and the assembly has a BUSCO v5.3.2 completeness of 95.6% (single = 92.5%, duplicated = 3.1%), using the laurasiatheria_odb10 reference set ( *n* = 12,234).

Metadata for specimens, barcode results, spectra estimates, sequencing runs, contaminants and pre-curation assembly statistics are given at https://tolqc.cog.sanger.ac.uk/darwin/mammals/Myotis_daubentonii/.

## Methods

### Sample acquisition and nucleic acid extraction

The specimen used for DNA sequencing was a male *M. daubentonii* (specimen ID SAN0001696, ToLID mMyoDau2) which was collected in Cologny, near Geneva, Switzerland (latitude 46.21, longitude 6.17) on 2017-06-23. It was found weakened on the ground and died shortly afterwards in captivity. Its body was preserved in ethanol in the collections of the Natural History Museum of Geneva under voucher number MHN-MAM-3002.100. The specimen was identified and dissected by Manuel Ruedi (Natural History Museum Geneva); tissue samples were immediately placed on dry ice before storage at –80°C.

The workflow for high molecular weight (HMW) DNA extraction at the Wellcome Sanger Institute (WSI) includes a sequence of core procedures: sample preparation; sample homogenisation, DNA extraction, fragmentation, and clean-up. In sample preparation, the mMyoDau2 sample was weighed and dissected on dry ice ( Jay *et al.*, 2023). For sample homogenisation, heart and muscle tissue was cryogenically disrupted using the Covaris cryoPREP ^®^ Automated Dry Pulverizer ( Narváez-Gómez *et al.*, 2023). HMW DNA was extracted using the Automated MagAttract v2 protocol ( Oatley *et al.*, 2023a). DNA was sheared into an average fragment size of 12–20 kb in a Megaruptor 3 system with speed setting 31 ( Bates *et al.*, 2023). Sheared DNA was purified by solid-phase reversible immobilisation ( Oatley *et al.*, 2023b): in brief, the method employs a 1.8X ratio of AMPure PB beads to sample to eliminate shorter fragments and concentrate the DNA. The concentration of the sheared and purified DNA was assessed using a Nanodrop spectrophotometer and Qubit Fluorometer and Qubit dsDNA High Sensitivity Assay kit. Fragment size distribution was evaluated by running the sample on the FemtoPulse system.

RNA was extracted from muscle tissue of mMyoDau2 in the Tree of Life Laboratory at the WSI using the RNA Extraction: Automated MagMax™ *mir*Vana protocol ( do Amaral *et al.*, 2023). The RNA concentration was assessed using a Nanodrop spectrophotometer and a Qubit Fluorometer using the Qubit RNA Broad-Range Assay kit. Analysis of the integrity of the RNA was done using the Agilent RNA 6000 Pico Kit and Eukaryotic Total RNA assay.

Protocols developed by the WSI Tree of Life laboratory are publicly available on protocols.io ( Denton *et al.*, 2023).

### Sequencing

Pacific Biosciences HiFi circular consensus DNA sequencing libraries were constructed according to the manufacturers’ instructions. Poly(A) RNA-Seq libraries were constructed using the NEB Ultra II RNA Library Prep kit. DNA and RNA sequencing was performed by the Scientific Operations core at the WSI on Pacific Biosciences SEQUEL II (HiFi) and Illumina NovaSeq 6000 (RNA-Seq) instruments. Hi-C data were also generated from muscle tissue of mMyoDau2 using the Arima2 kit and sequenced on the Illumina NovaSeq 6000 instrument.

### Genome assembly, curation and evaluation

Assembly was carried out with Hifiasm ( Cheng *et al.*, 2021) and haplotypic duplication was identified and removed with purge_dups ( Guan *et al.*, 2020). The assembly was then scaffolded with Hi-C data ( Rao *et al.*, 2014) using YaHS ( Zhou *et al.*, 2023). The assembly was checked for contamination and corrected as described previously ( Howe *et al.*, 2021). Manual curation was performed using HiGlass ( Kerpedjiev *et al.*, 2018) and PretextView ( Harry, 2022). The mitochondrial genome was assembled using MitoHiFi ( Uliano-Silva *et al.*, 2023), which runs MitoFinder ( Allio *et al.*, 2020) or MITOS ( Bernt *et al.*, 2013) and uses these annotations to select the final mitochondrial contig and to ensure the general quality of the sequence.

A Hi-C map for the final assembly was produced using bwa-mem2 ( Vasimuddin *et al.*, 2019) in the Cooler file format ( Abdennur & Mirny, 2020). To assess the assembly metrics, the *k*-mer completeness and QV consensus quality values were calculated in Merqury ( Rhie *et al.*, 2020). This work was done using Nextflow ( Di Tommaso *et al.*, 2017) DSL2 pipelines “sanger-tol/readmapping” ( Surana *et al.*, 2023a) and “sanger-tol/genomenote” ( Surana *et al.*, 2023b). The genome was analysed within the BlobToolKit environment ( Challis *et al.*, 2020) and BUSCO scores ( Manni *et al.*, 2021; Simão *et al.*, 2015) were calculated.

Table 3 contains a list of relevant software tool versions and sources.

**Table 3.** Software tools: versions and sources.

Software tool	Version	Source
BlobToolKit	4.2.1	https://github.com/blobtoolkit/blobtoolkit
BUSCO	5.3.2	https://gitlab.com/ezlab/busco
Hifiasm	0.16.1-r375	https://github.com/chhylp123/hifiasm
HiGlass	1.11.6	https://github.com/higlass/higlass
Merqury	MerquryFK	https://github.com/thegenemyers/MERQURY.FK
MitoHiFi	3	https://github.com/marcelauliano/MitoHiFi
PretextView	0.2	https://github.com/wtsi-hpag/PretextView
purge_dups	1.2.5	https://github.com/dfguan/purge_dups
sanger-tol/genomenote	v1.0	https://github.com/sanger-tol/genomenote
sanger-tol/readmapping	1.1.0	https://github.com/sanger-tol/readmapping/tree/1.1.0
YaHS	1.2a.2	https://github.com/c-zhou/yahs

### Wellcome Sanger Institute – Legal and Governance

The materials that have contributed to this genome note have been supplied by a Darwin Tree of Life Partner. The submission of materials by a Darwin Tree of Life Partner is subject to the **‘Darwin Tree of Life Project Sampling Code of Practice’**, which can be found in full on the Darwin Tree of Life website here. By agreeing with and signing up to the Sampling Code of Practice, the Darwin Tree of Life Partner agrees they will meet the legal and ethical requirements and standards set out within this document in respect of all samples acquired for, and supplied to, the Darwin Tree of Life Project.

Further, the Wellcome Sanger Institute employs a process whereby due diligence is carried out proportionate to the nature of the materials themselves, and the circumstances under which they have been/are to be collected and provided for use. The purpose of this is to address and mitigate any potential legal and/or ethical implications of receipt and use of the materials as part of the research project, and to ensure that in doing so we align with best practice wherever possible. The overarching areas of consideration are:

•     Ethical review of provenance and sourcing of the material

•     Legality of collection, transfer and use (national and international) 

Each transfer of samples is further undertaken according to a Research Collaboration Agreement or Material Transfer Agreement entered into by the Darwin Tree of Life Partner, Genome Research Limited (operating as the Wellcome Sanger Institute), and in some circumstances other Darwin Tree of Life collaborators.

## Data Availability

European Nucleotide Archive: *Myotis daubentonii* (Daubenton's bat). Accession number PRJEB61137; https://identifiers.org/ena.embl/PRJEB61137 ( Wellcome Sanger Institute, 2023). The genome sequence is released openly for reuse. The *Myotis daubentonii* genome sequencing initiative is part of the Darwin Tree of Life (DToL) project, the Bat1K Project and the Vertebrate Genomes Project (VGP). All raw sequence data and the assembly have been deposited in INSDC databases. The genome will be annotated using available RNA-Seq data and presented through the Ensembl pipeline at the European Bioinformatics Institute. Raw data and assembly accession identifiers are reported in Table 1.

## Author information

Members of the Darwin Tree of Life Barcoding collective are listed here: https://doi.org/10.5281/zenodo.4893703.

Members of the Wellcome Sanger Institute Tree of Life Management, Samples and Laboratory team are listed here: https://doi.org/10.5281/zenodo.10066175.

Members of Wellcome Sanger Institute Scientific Operations: Sequencing Operations are listed here: https://doi.org/10.5281/zenodo.10043364.

Members of the Wellcome Sanger Institute Tree of Life Core Informatics team are listed here: https://doi.org/10.5281/zenodo.10066637.

Members of the Tree of Life Core Informatics collective are listed here: https://doi.org/10.5281/zenodo.5013541.

Members of the Darwin Tree of Life Consortium are listed here: https://doi.org/10.5281/zenodo.4783558.
