# Supplementary material for: The genome sequence of Daubenton’s bat, Myotis daubentonii (Kuhl, 1817)
Source: Wellcome Open Res. 2024 Mar 1;9:107. [Version 1] doi: 10.12688/wellcomeopenres.21081.1 (PMC11252650; doi:10.12688/wellcomeopenres.21081.1)
Supplement: Peer review history

# Reviewer report: Reviewer response for version 1

**DOI:** 10.21956/wellcomeopenres.23320.r85172

**Published:** 2024-07-16

**Authors:** Diana D. Moreno Santillan

**Affiliations:** 1 University of California Berkeley, Berkeley, California, USA

The authors have presented a high-quality genome for *Myotis daubentonii*. The methods are thoroughly described and adhere to all standard protocols necessary for generating a high-quality genome. This data will be invaluable for researchers conducting comparative analyses in bats and other mammals, particularly in fields such as ecology, immunology, longevity, and other areas of significance.

Are sufficient details of methods and materials provided to allow replication by others?

Yes

Is the rationale for creating the dataset(s) clearly described?

Yes

Are the datasets clearly presented in a useable and accessible format?

Yes

Are the protocols appropriate and is the work technically sound?

Yes

Reviewer Expertise:

Molecular biology, Evolutionary Biology, Inflammaging, Immunity and longevity

I confirm that I have read this submission and believe that I have an appropriate level of expertise to confirm that it is of an acceptable scientific standard.

# Reviewer report: Reviewer response for version 1

**DOI:** 10.21956/wellcomeopenres.23320.r87674

**Published:** 2024-07-16

**Authors:** Attila D Sándor

**Affiliations:** 1 University of Veterinary Medicine, Budapest, Hungary

This is a brief note providing details for the genome sequence of a Daubenton's bat from Central Europe. It presents the collection details, methodology and assessment of the genome, providing free access to the coded data, too. While the text is brief, it contains all the denominators one may need while accessing the data. The rationale is sound and the data will undoubtedly be a major gateway for further studies.

 I have two minor details to highlight:

- Introduction lists a series of information on the species, including morphology and ecology. If those are not the author's own observations (highly unlikely, based on a single animal), a reference would be welcome.
- In addition, the issued weight range (6-10 g) covers less than 80 of the range I observed on the upper limit, I suggest to use 6-14 g.

Are sufficient details of methods and materials provided to allow replication by others?

Yes

Is the rationale for creating the dataset(s) clearly described?

Yes

Are the datasets clearly presented in a useable and accessible format?

Yes

Are the protocols appropriate and is the work technically sound?

Yes

Reviewer Expertise:

Zoologist, primarily working in vertebrate ecology, parasitology and biogeography, targeting the importance of genetic background in host-parasite-pathogen cycles, using bats and their parasites as model.

I confirm that I have read this submission and believe that I have an appropriate level of expertise to confirm that it is of an acceptable scientific standard.

# Reviewer report: Reviewer response for version 1

**DOI:** 10.21956/wellcomeopenres.23320.r85171

**Published:** 2024-06-13

**Authors:** Jorge Ortega

**Affiliations:** 1 Instituto Politecnico Nacional, Mexico City, Mexico City, Mexico

The authors do an exceptional job describing the major components of the Myotis daubentonii genome. In general, there are not many discrepancies with previously published works with respect to results and methodology. Below I would like to clarify some particular points: 1) they could confirm the correct identity (characters considered) of the specimen because the literature reports possible hybridization with other Myotis species, so being a relatively abundant and widely distributed species there may be some confusion; 2) they could talk a little about the isolation by distance that the species presents in its distribution (Laine et al. 2003), which could mean a complex of cryptic species without forming a monophyletic clade (Kawai et al. 2003); 3) it would be good to consider including bibliography, not so recent (Bogdanowicz 1994), but that describes much of the natural history of the species; 4) the species is considered least concern for some European countries, but in danger of extinction for others such as Austria and Germany; 5) justify why they used two specimens for the analyses.

Are sufficient details of methods and materials provided to allow replication by others?

Yes

Is the rationale for creating the dataset(s) clearly described?

Yes

Are the datasets clearly presented in a useable and accessible format?

Yes

Are the protocols appropriate and is the work technically sound?

Yes

Reviewer Expertise:

Immunogenetics. Population genetics. Genomics.

I confirm that I have read this submission and believe that I have an appropriate level of expertise to confirm that it is of an acceptable scientific standard.
